# The Conundrum of Tricuspid Regurgitation Grading

**DOI:** 10.3389/fcvm.2018.00164

**Published:** 2018-11-09

**Authors:** Yun Yun Go, Raluca Dulgheru, Patrizio Lancellotti

**Affiliations:** ^1^National Heart Centre Singapore, National Heart Research Institute Singapore, Singapore, Singapore; ^2^Department of Cardiology, University Hospital Sart Tilman, Heart Valve Clinic, University of Liège, Liège, Belgium; ^3^GIGA Cardiovascular Sciences, University Hospital Sart Tilman, Liège, Belgium; ^4^Gruppo Villa Maria Care and Research, Anthea Hospital, Bari, Italy

**Keywords:** tricuspid regurgitation grading, massive tricuspid regurgitation, torrential tricuspid regurgitation, echocardiography, percutaneous intervention

## Abstract

Findings from early percutaneous tricuspid intervention trials have shown that the severity of tricuspid regurgitation (TR) far exceeded the current definition of severe TR. Also, the improvement in the amount of TR following tricuspid intervention is not accounted for by the current definition of TR as different degrees of severity at the severe end was grouped under the same umbrella term of “severe.” There has been a recent call to expand the TR grading system, encompassing two more grades, namely “massive” and “torrential” TR, in the order of increasing severity. This seems appropriate as the patients enrolled in tricuspid intervention trials were found to have TR severity up to 2 grades above the current severe thresholds of effective regurgitant orifice area (EROA) 40 mm^2^, regurgitant volume (R Vol) 45 ml and vena contracta (VC) width 7 mm. The proposed grade of “massive” is defined by EROA 60–79 mm^2^, R Vol 60–74 ml and VC 14–20 mm, while “torrential” is defined by EROA ≥80 mm^2^, R Vol ≥75 ml, and VC ≥21 mm. The grading of TR requires a comprehensive, multi-parametric approach. In particular, quantitative assessment of TR should be performed in patients who require serial monitoring and quantification of treatment effect.

## Introduction

The tricuspid valve has often been labeled the forgotten valve, due to the lack of attention it received compared to its counterparts such as the mitral or aortic valve (1). Research interests and advancement in therapy, most notably percutaneous valve intervention for years seems to elude the tricuspid valve. In recent years, the tide appears to be turning. We witnessed a rise in interest and developmental breakthroughs in the treatment of functional or secondary tricuspid regurgitation (TR). The movement is both appropriate and timely as functional TR is a common but often-overlooked clinical problem. Severe TR is associated with increased morbidity and mortality (2, 3). Despite the poor outcomes, the number of patients with severe TR undergoing tricuspid valve surgery was dismal. Approximately 1.6 million patients in the United States live with moderate to severe TR and there are <8,000 tricuspid surgery performed annually (4, 5). The overwhelming majority of tricuspid valve surgery was performed during concomitant left-sided valve surgery (6). Among patients who underwent left-sided valve surgery, 37% eventually developed severe TR following rheumatic mitral valve replacement and 74% had moderate to severe TR 3 years after ischemic mitral repair surgery (7, 8). This not only speaks volume of the unmet need for TR treatment, but also illustrates the treatment difficulty in patients with previous left-sided heart surgery who need downstream TR intervention, either in the form of redo surgery or percutaneous therapy. Last but not least, it highlights the importance of comprehensive and methodical echocardiographic assessment prior to or following any valve surgery.

## Echocardiographic assessment of TR

Echocardiography is the imaging modality of choice to assess TR severity and in turn, help to guide decision for treatment. The guideline-recommended echocardiographic assessment of TR is, however, not without its challenges. It involves a multi-parametric approach and calls for qualitative, semi-quantitative, and quantitative evaluation (9). Qualitative and semi-quantitative assessment are relatively straightforward and intuitive as they do not require multi-step calculations as quantitative approach does. As a result, many clinicians often adopt the qualitative or semi-quantitative approach, which give rise to two problems. First, it renders most comparisons, either serially over time or between patients difficult due to the lack of precision and standardization. Second, it is prone to under-estimation of the regurgitant severity.

Traditionally, TR is graded into mild, moderate, and severe, following the severity grading conventions that also apply to mitral, aortic, and pulmonary valves. Severe TR is defined quantitatively as an effective regurgitant orifice area (EROA) of ≥40 mm^2^ and a regurgitant volume of ≥45 ml according to both the European Association of Cardiovascular Imaging (EACVI) and American Society of Echocardiography (ASE) recommendations (9, 10). In particular, the EACVI recommendations made provision for massive TR. Massive TR is referred to as TR that is beyond severe and it is associated with a low TR jet velocity of <2 m/s as there is near equalization of right ventricular and right atrial pressures (9). However, in the EACVI recommendations, massive TR was defined qualitatively. There were no quantitative or semi-quantitative parameters that distinguish massive from severe TR and therein lies a 2-fold problem. For starter, the distinction between severe and massive is not always clear. Qualitative parameters such as the size of the TR jet on color Doppler can change substantially according to the Nyquist limits and loading conditions. Similarly, in the case of massive TR with very large regurgitant orifice area, a distinct color jet may not be apparent due to the presence of laminar, low velocity flow (10). Also, the use of descriptive terminology without proper standardization and quantification lacks the scientific rigor necessary for quality research. Terms such as very severe, free, massive, or torrential are descriptive and thus subjected to interpretation. Loose application or interchangeable usage of these terms are not uncommon both the published literature and clinical practice, making meaningful, quantitative comparisons impossible.

“Of note, the EACVI guidelines do not recommend EROA calculation using the quantitative PW Doppler method due to the lack of evidence that supports its use in TR quantification. The quantitative Doppler method however, has been used in mitral regurgitation studies and it systematically produces a larger EROA compared to PISA-derived EROA (11). Whether these findings can be extrapolated in patients with TR and if the cut-offs for severity grading are comparable warrant further studies.”

## Revision of the current TR grading system

In recent times, there has been a movement to revise the current TR grading, expanding the TR grading spectrum beyond severe to include “massive” and “torrential,” in ascending severity (12). There are a few impetuses to expand the existing guideline-recommended TR grading. First, the rise of percutaneous tricuspid valve intervention trials and registry saw the enrolment of patients with TR far exceeded the severe limit defined by current guidelines (13). These patients had on average, TR with VC width, EROA or regurgitant volume one to two grades above the current definition of severe. Such magnitude calls to question the adequacy of the current definition at depicting the complete clinical picture. The lack of further distinction at the extreme end of the TR spectrum not only leads to non-discriminatory treatment of TR with different severity and prognosis, but also makes treatment effect difficult to detect. Natural history studies have shown that patients' prognosis worsened as the severity of TR increases, supporting the case for grading TR beyond severe to reflect the differential outcomes. This is particularly relevant among patients who received percutaneous TV interventions. After all, it is challenging to explain to patients that a therapy which improves their TR from severe to severe might still benefit them.

Some percutaneous TR intervention trials that included sick patients with torrential TR reported improvement in TR parameters equivalent to one full grade reduction or 20 mm^2^ in terms of EROA (13, 14). There was also improvement in measurable clinical outcomes such as New York Heart Association (NYHA) class, quality of life and 6-min walk test. Although there was a lack of head-to-head comparison with patients treated with medical therapy alone in these trials, it is not unreasonable to associate these improvements with the reduction of EROA, reduction in tricuspid annulus size and increase in forward stroke volume, as a result of the intervention.

In the SCOUT (Percutaneous Tricuspid Valve Annuloplasty System for Symptomatic Chronic Functional Tricuspid Regurgitation) trial, the average quantitative EROA of the cohort was 85 mm^2^ (13), which is more than 2 grades above the existing severe threshold of 40 mm^2^, assuming a grade difference of 20 mm^2^. Also, the average vena contracta (VC) of the SCOUT cohort was 13 mm, which is ~2 grades above the existing severe threshold of 7 mm, assuming a grade difference of 3–4 mm. The transcatheter plication system produced a reduction of quantitative EROA of >20 mm^2^, more than a full grade reduction of TR, which was both statistically significant and clinically meaningful. In the International Multicentre TriValve Registry, the average tricuspid EROA was 87 ± 56 mm^2^ and the average regurgitant volume was 63 ml, while the average VC was 11 mm (14), all at 1–2 grades above the current thresholds for severe TR, defined as EROA of 40 mm^2^, regurgitant volume of 45 ml and VC of 7 mm.

The argument for the expansion of the current TR grading system stems primarily, although not solely, from the growing need to standardize and quantify transcatheter tricuspid treatment effect. With no specific criteria that capture the disease severity at the tail end of the TR spectrum, there is a risk of diluting measurable treatment effect. Currently, procedural success is defined as residual TR of ≤2+, successful implantation of device and patients being alive at the end of the procedure, similar to the definition used in mitral valve intervention. Only 62% of patients in the International Multicentre TriValve Registry achieved procedural success by definition and 51% of patients still had ≥3+ TR on discharge echocardiography. There was however, significant improvement in NYHA functional class and reduction in diuretic requirement at 30 days (14). The absence of a detailed grading system to quantify treatment effect may account for the discrepancy between the lack of significant TR grade reduction and the improvement of quality of life following successful percutaneous TV intervention.

It is inevitable that an improved TR grading system will be needed in view of the inadequacy of current definition. Table 1 illustrates a proposed grading system, with emphasis on a systematic, multi-parametric approach. Table 2 summarizes the improvements made in the new grading system compared to the current guidelines. It must be mentioned that the proposed TR grading system is based mainly on data gathered from percutaneous tricuspid intervention studies, which tend to be modest in study size and highly selective. The grading system therefore requires further refinement and support from large natural history, registry or outcome studies.

**Table 1:** Proposed tricuspid regurgitation grading.

	**Mild**	**Moderate**	**Severe**	Massive^*^	Torrential^*^
**QUALITATIVE**					
TV morphology	Normal/abnormal	Normal/abnormal	Abnormal/flail/large coaptation defect		
Color Doppler of TR jet	Small, central	Intermediate	Very large central jet or eccentric wall impinging jet		
CW signal of TR jet	Faint/parabolic	Dense/parabolic	Dense/triangular with early peaking	Peak TR velocity < 2 m/s	—
**SEMIQUANTITATIVE**					
VC width (mm)^§^	< 3	3–6.9	7–13.9	14–20	>21
PISA radius (mm)	≤ 5	6–9	>9	—	—
Hepatic vein flow	Systolic dominance	Systolic blunting	Systolic flow reversal		
Tricuspid inflow	Normal	Normal	E wave dominant (≥1 cm/s)		
**QUANTITATIVE**					
EROA (mm^2^) by PISA	< 20	20–39	40–59	60–79	≥80
EROA (mm^2^) by quantitative Doppler	—	—	75–94	95–114	≥115
EROA (mm^2^) by 3D	–	–	75–94	95–114	≥115
R Vol (ml) by PISA	< 30	30–44	45–59	60–74	≥75

*TV, tricuspid valve; TR, tricuspid regurgitation; CW, continuous wave; VC, vena contracta; PISA, proximal isovelocity surface area; EROA, effective regurgitant orifice area; R Vol, regurgitant volume*.

* *further data required*.

§ *preferably biplane*.

**Table 2:** Comparisons of current guideline vs. proposed changes to TR grading.

**Proposed TR grading**	**Current guidelines**
“Massive” for TR one grade above severe and “torrential” for the most severe form of TR possible	Made provision for massive TR qualitatively. No clear semi-quantitative or quantitative definition
Different thresholds for EROA obtained from PISA and quantitative Doppler methods	No distinction between EROA obtained from PISA and quantitative Doppler
EROAs and regurgitant volumes for massive and torrential TR were defined.	Only thresholds for severe (EROA ≥40 mm^2^ and R Vol ≥45 ml) were defined.
Use of 3D vena contracta/effective regurgitant orifice area (the resultant value should be comparable to EROA obtained from quantitative Doppler)	No mention of 3D assessment
Use of biplane vena contracta	Did not emphasise the use of biplane vena contracta

“In summary, there is no easy solution to the TR grading conundrum. Perhaps one that come close is to place heavier emphasis on quantitative assessment of TR. In the context of tricuspid valve intervention, this can be carried out by routinely report the EROA before and after the procedure in terms of absolute or percentage change. An alternative, as proposed by Han et al. would be to expand the current grading system linearly by adding two more grades beyond the current definition of severe TR. More data, especially long-term outcome data is needed to resolve the conundrum.”

## Conclusion

TR has a wide disease spectrum, especially toward the severe end. A grading approach that emphasize on quantitative assessment account for the granularity of disease and give equal weightage to the full disease spectrum. It incorporates our new understanding of baseline TR severity and make allowance for measurable TR reduction in the era of percutaneous tricuspid intervention. It also introduces checks and balances, moving from subjective definition to a standardized lingo. In the foreseeable future, it is likely to have an influence on and in turn, be influenced by the design and evaluation of tricuspid interventional trials.

## Author contributions

All authors listed have made a substantial, direct and intellectual contribution to the work, and approved it for publication.

### Conflict of interest statement

The authors declare that the research was conducted in the absence of any commercial or financial relationships that could be construed as a potential conflict of interest. The reviewer NB and handling Editor declared their shared affiliation.
